# Associations between Mediterranean lifestyle and its components with cardiovascular outcomes among community-dwelling older adults

**DOI:** 10.3389/fepid.2026.1933837

**Published:** 2026-10-21

**Authors:** Hélio José Coelho-Júnior, Emanuele Marzetti

**Affiliations:** 1 Fondazione Policlinico Universitario Agostino Gemelli IRCCS, Rome, Italy; 2 Department of Geriatrics, Orthopedics and Rheumatology, Università Cattolica Del Sacro Cuore, Rome, Italy

**Keywords:** diet, hypertension, myocardial infarction, nutritional habits, physical activity, stroke

## Abstract

**Aim:**

The present study examined the cross-sectional and longitudinal associations of adherence to the Mediterranean lifestyle (MEDLS) and its individual components with cardiovascular outcomes among older adults in England.

**Methods:**

Data from 565 adults aged ≥65 years participating in the English Longitudinal Study of Ageing were analyzed. MEDLS adherence (score range: 0–24) comprised six domains: Mediterranean diet adherence, physical activity, water intake, sleep, social isolation, and cooking activities. The outcomes of interest were hypertension, heart attack (myocardial infarction/coronary thrombosis), and stroke. Logistic regression models were used to examine cross-sectional and longitudinal associations, with analyses adjusted for age and sex.

**Results:**

Among 565 participants, 96 had hypertension, 14 had experienced a heart attack, and 10 had experienced a stroke at baseline; 42, 16, and 13 incident cases, respectively, were identified during follow-up. Higher MEDLS adherence was cross-sectionally associated with lower odds of hypertension (OR 0.849, 95% CI 0.722–0.999). No statistically significant longitudinal associations were observed between MEDLS and hypertension (OR 0.990, 95% CI 0.902–1.085), heart attack (OR 0.919, 95% CI 0.707–1.195), or stroke (OR 1.053, 95% CI 0.827–1.341), although the small number of events limited precision. At the component level, Mediterranean diet adherence was associated with lower odds of incident heart attack (OR 0.670, 95% CI 0.454–0.988); However, this finding was considered exploratory.

**Conclusion:**

Higher adherence to the MEDLS was associated with lower odds of prevalent hypertension but not with incident cardiovascular outcomes among communitydwelling older adults. At the component level, greater adherence to the Mediterranean diet was prospectively associated with lower odds of heart attack; however, given the absence of adjustment for multiple comparisons, this finding should be considered exploratory and requires confirmation in adequately powered studies.

## Introduction

The Mediterranean (MED) diet is a traditionally defined dietary pattern that emerged from millennial cultural exchanges among populations inhabiting the regions surrounding the MED basin (1, 2). It is characterized by high consumption of fruits, vegetables, whole grains, legumes, nuts, and olive oil, moderate intake of fish; occasional consumption of alcohol usually during meals, mainly wine; and limited intake of red meat and processed foods (1, 2). The MED diet gained substantial scientific attention in the 1990s, when population-based cohort studies reported a reduced risk of mortality among individuals with higher adherence (3, 4). Since then, numerous investigations have confirmed and expanded these findings, extending the benefits of the MED diet to a wide range of health outcomes (5–10).

Particularly, accumulating evidence suggests that greater adherence to the MED diet promotes cardiovascular health by reducing the risk of major cardiovascular events, including coronary heart disease and ischemic stroke, while also improving cardiometabolic risk factors such as blood pressure, lipid profile, glycemic control, inflammation, and endothelial function. These benefits may collectively contribute to a slower age-related decline in cardiovascular health (11, 12).

More recently, this concept has been expanded in response to arguments that the functional benefits attributed to the MED diet may stem not only from dietary intake itself but also from its integration into a broader MED way of living (2, 13). This lifestyle is traditionally characterized by high levels of physical activity (PA), strong social engagement, and adequate sleep, among other behaviors strongly associated with better health outcomes (14–20). Such reasoning led to the emergence of the term Mediterranean lifestyle (MEDLS) (2, 13).

Since then, an increasing number of studies have examined the effects of the MEDLS framework (21–26). Nonetheless, the application of this framework outside the MED basis has been examined only sparingly. Based on these premises, the present study examined data from the English Longitudinal Study of Ageing (ELSA) to provide a comprehensive assessment of the cross-sectional and longitudinal associations between MEDLS and its components with hypertension, heart attack (myocardial infarction/coronary thrombosis), and stroke among community-dwelling older adults.

## Methods

### Study design and population

The ELSA is a longitudinal cohort of adults aged ≥50 years in England, with repeated assessments of physical performance, cognition, health behaviors, chronic conditions, and biological markers (27). The study began in 2002–2003, and the most recent data collection occurred in Wave 11 (2023–2024). Nutritional information, including detailed dietary intake, was introduced in Wave 9 (2018–2019), which therefore served as the baseline for the present analysis. Wave 11 (2023–2024) served as the follow-up point, providing approximately 6 years of longitudinal observation.

For this study, we restricted the analytical sample to participants aged ≥65 years at baseline. Individuals using mobility aids (i.e., manual or electric wheelchair, buggy, or scooter) and those with major chronic conditions at baseline (cancer, Parkinson's disease, psychiatric disorders, Alzheimer's disease, multiple sclerosis, or motor neuron disease) were excluded. Participants who developed these conditions during follow-up and those who had COVID-19-related hospitalization were also excluded from longitudinal analyses.

Regarding nutritional information, 6,730 participants were excluded due to missing Food Frequency Questionnaire (FFQ) data. Among those with available FFQ data, 1,880 were excluded because of insufficient information to compute the Medi-Lite score. Participants lacking sufficient data to compute the overall MEDLS score were also excluded, except for sleep duration, which was retained despite 222 missing values.

The ELSA study adhered to the ethical principles outlined in the Declaration of Helsinki and received ethical approval from the National Research Ethics Service (MREC/01/2/91). Informed consent was obtained from each participant before data collection. The study is reported in accordance with the Strengthening the Reporting of Observational Studies in Epidemiology (STROBE) guidelines (28).

#### Mediterranean lifestyle

Adherence to MEDLS was estimated using an adapted version of the index proposed by the Department of Preventive Medicine and Public Health of the School of Medicine of the Universidad Autónoma de Madrid, Madrid, Spain (21). The MEDLS index used in the present study comprises a combination of 17 variables—nine MED diet components and eight lifestyle or social behavior items (2, 13), including (i) dietary habits, (ii) PA levels, (iii) hydration status, (iv) social isolation, (v) sleeping patterns, and (vi) cooking activities. The nine dietary components contributed up to 18 points, whereas the lifestyle and social components contributed up to 6 additional points: PA (0–1), hydration (0–1), social isolation (0–2), sleeping patterns (0–1), and cooking activities (0–1). Individual components were summed to generate the total MEDLS score (range 0–24), with higher scores indicating greater adherence to the Mediterranean lifestyle. The scoring criteria and contribution of each component to the total MEDLS are presented in Table 1. This index was chosen because it captures the multidimensional nature of MEDLS (diet, physical activity, rest, and social habits) and has demonstrated construct validity in epidemiological populations.

**Table 1:** Scoring system used to calculate the Mediterranean lifestyle score (MEDLS).

MEDLS component	Scoring criteria	Points
Mediterranean diet (Medi-Lite)	Nine dietary components, each scored from 0 to 2 according to Medi-Lite criteria	0–18
Physical activity	≥600 MET·min/week = 1; <600 MET·min/week = 0	0–1
Hydration status	≥5 glasses of water/day = 1; <5 glasses/day = 0	0–1
Social isolation	Low social isolation = 2; moderate social isolation = 1; high social isolation = 0	0–2
Sleeping patterns	6–8 h/day = 1; <6 or >8 h/day = 0	0–1
Cooking activities	Regular preparation and cooking of meals at home = 1; otherwise = 0	0–1
Lifestyle and social components	Sum of physical activity, hydration, social isolation, sleeping patterns, and cooking activities	0–6
Total MEDLS	Medi-Lite score + lifestyle and social components	0–24

Higher MEDLS scores indicate greater adherence to the Mediterranean lifestyle.

Adherence to the MED diet was assessed using the Medi-Lite score (0–18) (29, 30), calculated across nine food categories. Dietary intake was assessed using the FFQ administered in Wave 9 of the ELSA via the Oxford web questionnaire. A score from 0 (lowest intake) to 2 (highest consumption) was assigned to food items usually included in a Medi-Diet (i.e., cereals, fruits, legumes, fish, and vegetables). For dietary categories not included in a typical Medi-Diet (e.g., meat and dairy products), 2 points were allocated to the lowest intake, 1 point to an intermediate consumption, and 0 points to the highest consumption. For olive oil, 2 points were assigned for daily usage, 1 point for regular use, and 0 points for occasional consumption. For alcohol consumption, 1 alcohol unit was defined as 12 g of alcohol. Consumption of >0 to <1 unit/day received 1 point, whereas consumption of 1–2 units/day received 2 points. Abstinence and consumption of >2 units/day received 0 points. Overall scores ranged from 0 to 18, with higher scores indicating greater adherence to the MED diet.

PA levels, expressed as metabolic equivalent task (MET)·min/week, were estimated from questionnaire data based on participants' responses to PA items. At baseline, participants reported how often they engaged in vigorous, moderate, and light physical activities, selecting one of the following options: *more than once per week*, *once per week*, *one to three times per month*, or *hardly ever*. PA levels were then dichotomized as high (≥600 MET·min/week) or low (<600 MET·min/week) (31). Participants with high PA levels received 1 point, and those with low PA levels received 0 points.

Hydration status was assessed based on participants' reported intake of water consumed from a glass or beaker (e.g., still, sparkling, or tap water). Adequate hydration was defined as consuming ≥5 glasses per day, corresponding to approximately 50% of the daily fluid intake typically recommended for older adults. Participants meeting this criterion received 1 point, while those consuming <5 glasses per day received 0 points.

Social isolation was assessed using items evaluating feelings of lacking companionship and perceived isolation from others. Response options included: hardly ever or never, some of the time, and often. Participants who reported feeling a lack of companionship or isolation some of the time or often were classified as socially isolated. Participants with low social isolation received 2 points, those with moderate social isolation received 1 point, and those with high social isolation received 0 points. Higher scores therefore indicated lower social isolation. These parameters represent complementary social constructs: social isolation refers to structural social deprivation, while loneliness is linked to subjective emotional distress (17).

Sleep patterns were assessed by calculating the total hours slept during 24 h preceding data collection. Participants who reported sleeping between 6 and 8 h were classified as having adequate sleep duration and received 1 point; otherwise, 0 points were assigned (21).

Cooking activity was assessed using a questionnaire item asking participants whether they regularly prepared and cooked meals at home. Responses were categorized as yes/no, with 1 point assigned to participants who reported regular engagement in meal preparation and cooking and 0 points to those who did not.

#### Cardiovascular outcomes

Incidence of cardiovascular diseases was defined based on self-reported new physician diagnoses since the previous interview, including hypertension (variable = hediia1), heart attack (including myocardial infarction or coronary thrombosis; variable = hediia3), and stroke (variable = hediia7).

#### Covariates

Age was recorded in years, and sex was documented as male or female.

#### Statistical analysis

Continuous variables were expressed as means ± standard deviations, and categorical variables as %. Logistic regression models were used to examine associations between MEDLS and hypertension, heart attack, and stroke. Given the limited number of cardiovascular events, adjusted models were parsimoniously specified to reduce the risk of overfitting, in line with the traditional recommendation of approximately 10 events per estimated parameter. Accordingly, the adjusted models included age and sex as covariates. Other potentially relevant cardiovascular risk factors could not be included because the number of available events was insufficient to support more extensively adjusted models. To identify which MEDLS components contributed to the observed significant associations, each aspect was analyzed individually. Longitudinal analyses were conducted after excluding participants with the corresponding cardiovascular condition at baseline. No statistical imputation was performed for missing sleep data. Participants with missing sleep information were retained, with the sleep component contributing 0 points to the total MEDLS score. Effect sizes and 95% confidence intervals were calculated for all analyses, and significance was set at *p* < 0.05. Analyses were conducted using SPSS version 23 (IBM, New York, NY, USA).

## Results

### Characteristics of participants

The main characteristics of the study participants are presented in Table 2 (*n* = 565). Participants had a mean age of 76.2 ± 7.8 years, and most were women (55.8%). The MEDLS had a theoretical range of 0–24 points and an observed range of 4–17 points in the present sample. The Medi-Lite dietary component had a theoretical range of 0–18 points and an observed range of 4–14 points. Feelings of social isolation were highly prevalent among participants (81.8%). Cooking activities were reported by 56.8% of participants. In addition, 33.6% met PA recommendations, 95.6% slept for 6–8 h, and 6.5% maintained adequate hydration levels.

**Table 2:** Main characteristics of study participants (*n* = 565).

Variables	Values
Age (years)	76.2 (7.8)
Female, *n* (%)	315 (55.8)
Caloric intake (kcal/day)	2,106.82 (715.94)
Current smoking, *n* (%)	53 (9.4)
MEDLS (0–24), mean (SD)	11.03 (2.20)
Medi-Lite dietary score (0–18), mean (SD)	9.36 (1.94)
Physical activity (≥600 MET·min/day), *n* (%)	190 (33.6)
Hydration status (≥5 glasses per day), *n* (%)	37 (6.5)
Presence of social isolation, *n* (%)	462 (81.8)
Sleeping patterns (6–8 h), *n* (%)	540 (95.6)
Cooking activities (yes), *n* (%)	321 (56.8)
Prevalence of hypertension, *n* (%)	96 (17.0)
Prevalence of heart attack, *n* (%)	14 (2.5)
Prevalence of stroke, *n* (%)	10 (1.8)
Incidence of hypertension, *n* (%)	42 (7.4)
Incidence of heart attack, *n* (%)	16 (2.8)
Incidence of stroke, *n* (%)	13 (2.3)

Data are presented as mean (SD) or *n* (%).

Incident cases were identified after excluding participants with the corresponding cardiovascular condition at baseline. Percentages were calculated using the total analytical sample (*n* = 565).

### Cross-sectional and longitudinal associations between MEDLS and cardiovascular conditions

The cross-sectional and longitudinal associations between MEDLS and cardiovascular outcomes are presented in Table 3. In the adjusted cross-sectional analysis, each one-point increase in MEDLS was associated with lower odds of hypertension (OR 0.849, 95% CI 0.722–0.999; *p* = 0.048). The adjusted cross-sectional MEDLS–heart attack model could not be reliably estimated because of sparse outcome data, while no association was observed with stroke (OR 1.302, 95% CI 0.754–2.249; *p* = 0.344). In the adjusted longitudinal analyses, MEDLS was not significantly associated with incident hypertension (OR 0.990, 95% CI 0.902–1.085; *p* = 0.824), heart attack (OR 0.919, 95% CI 0.707–1.195; *p* = 0.538), or stroke (OR 1.053, 95% CI 0.827–1.341; *p* = 0.676). For contrast, the inverse unadjusted associations with heart attack observed cross-sectionally (OR 0.712, 95% CI 0.523–0.963; *p* = 0.027) and longitudinally (OR 0.808, 95% CI 0.671–0.973; *p* = 0.024) were not supported after covariate adjustment.

**Table 3:** Binary longitudinal regressions between MEDLS and cardiovascular conditions.

	Cross-sectional						Longitudinal					
	Unadjusted			Adjusted			Unadjusted			Adjusted		
Variables	OR	95% CI	*p*-Value	OR	95% CI	*p*-Value	OR	95% CI	*p*-Value	OR	95% CI	*p*-Value
Hypertension	0.929	0.853, 1.012	0.093	**0.849**	**0.722, 0.999**	**0.048**	0.934	0.872, 1.000	0.051	0.99	0.902, 1.085	0.824
Heart attack	**0.712**	**0.523, 0.963**	**0**.**027**	—	—	—	**0**.**808**	**0.671, 0.973**	**0**.**024**	0.919	0.707, 1.195	0.538
Stroke	0.935	0.730, 1.199	0.599	1.302	0.754, 2.249	0.344	0.931	0.731, 1.095	0.387	1.053	0.827, 1.341	0.676

Bold denotes significance. Longitudinal analyses were conducted after excluding participants with the corresponding cardiovascular condition at baseline.

OR,  odds ratio; CI, confidence interval; —, the model could not be reliably estimated because of sparse or empty outcome-by-exposure cells..

### Associations between MEDLS components and cardiovascular outcomes

The associations between the individual MEDLS components and cardiovascular outcomes are presented in Table 4. In the adjusted analyses, greater adherence to the Mediterranean diet was associated with lower odds of incident heart attack (OR 0.670, 95% CI 0.454–0.988; *p* = 0.044). Given its borderline confidence interval and the absence of adjustment for multiple comparisons, this finding should be interpreted as exploratory. No other component-specific adjusted association reached statistical significance, and several models could not be reliably estimated because of sparse or empty cells. For contrast, the unadjusted cross-sectional association between cooking activities and hypertension (OR 0.375, 95% CI 0.147–0.956; *p* = 0.040) did not persist after adjustment (OR 0.295, 95% CI 0.063–1.384; *p* = 0.122).

**Table 4:** Binary longitudinal regressions between MEDLS components and cardiovascular conditions.

	Cross-sectional						Longitudinal					
MEDLS components	Unadjusted			Adjusted			Unadjusted			Adjusted		
	OR	95% CI	*p*-Value	OR	95% CI	*p*-Value	OR	95% CI	*p*-Value	OR	95% CI	*p*-Value
Hypertension												
MED diet	0.892	0.656, 1.212	0.463	0.895	0.653, 1.229	0.494	0.939	0.789, 1.118	0.480	1.059	0.675, 1.661	0.805
Physical activity	0.980	0.476, 2.017	0.956	0.969	0.441, 2.126	0.937	0.955	0.587, 1.555	0.854	1.585	0.475, 5.295	0.454
Hydration status	—	—	—	—	—	—	0.870	0.196, 3.856	0.855	0.826	0.184, 3.702	0.803
Social isolation	0.878	0.677, 1.140	0.328	0.963	0.607, 1.529	0.875	0.931	0.762, 1.137	0.483	1.109	0.482, 2.552	0.807
Sleeping patterns	0.660	0.063, 6.958	0.730	0.746	0.067, 8.266	0.811	2.056	0.263, 16.042	0.492	2.316	0.295, 18.204	0.425
Cooking activities	**0.375**	**0.147, 0.956**	**0.040**	0.295	0.063, 1.384	0.122	0.870	0.527, 1.438	0.588	0.380	0.063, 2.295	0.292
Heart attack												
MED diet	**0.712**	**0.526, 0.963**	**0.027**	—	—	—	**0**.**623**	**0.418, 0.929**	**0**.**020**	**0**.**670**	**0.454, 0.988**	**0**.**044**
Physical activity	—	—	—	—	—	—	0.233	0.029, 1.871	0.170	0.231	0.028, 1.866	0.169
Hydration status	—	—	—	—	—	—	—	—	—	—	—	—
Social isolation	0.999	0.527, 1.893	0.996	1.061	0.548, 2.056	0.860	0.847	0.518, 1.383	0.507	0.804	0.489, 1.321	0.389
Sleeping patterns	—	—	—	—	—	—	0.173	0.018, 1.649	0.127	0.188	0.019, 1.866	0.154
Cooking activities	—	—	—	—	—	—	1.132	0.343, 3.739	0.838	1.208	0.350, 4.177	0.765
Stroke												
MED diet	0.799	0.378, 1.687	0.556	0.843	0.403, 1.763	0.650	1.046	0.665, 1.646	0.846	1.048	0.668, 1.645	0.838
Physical activity	4.000	0.358, 44.738	0.260	0.095	0.006, 1.535	0.097	1.580	0.478, 5.220	0.454	1.653	0.496, 5.506	0.413
Hydration status	—	—	—	—	—	—	—	—	—			
Social isolation	1.112	0.501, 2.471	0.794	2.006	0.310, 12.891	0.465	1.228	0.738, 2.043	0.430	1.193	0.724, 1.967	0.489
Sleeping patterns	—	—	—	—	—	—	—	—	—	—	—	—
Cooking activities	—	—	—	—	—	—	0.529	0.152, 1.841	0.317	0.550	0.156, 1.934	0.351

Bold denotes significance. Longitudinal analyses were conducted after excluding participants with the corresponding cardiovascular condition at baseline.

OR,  odds ratio; CI, confidence interval; —, the model could not be reliably estimated because of sparse or empty outcome-by-exposure cells.

## Discussion

The main findings of the present study suggest that high adherence to MEDLS was cross-sectionally, significantly, and inversely associated with hypertension. In contrast, no significant associations with MEDLS were observed in the longitudinal analysis. However, when the individual MEDLS components were examined, greater adherence to the Mediterranean diet was associated with lower odds of incident heart attack. Given the borderline confidence interval and the absence of correction for multiple comparisons, this finding should be interpreted cautiously as exploratory rather than causal.

There is a growing body of studies examining the associations between MEDLS and cardiovascular conditions, with investigations presenting mixed results. Talavera-Rodríguez et al. (22) observed a tendency toward lower systolic blood pressure values as adherence to MEDLS increased. Indeed, each two-point increment in the MEDLS score was associated with lower blood pressure values (22). Similar findings were reported by Sotos-Prieto et al. (32), who analyzed data of 11,090 individuals (aged ≥18 years) from the Study on Nutrition and Cardiovascular Risk in Spain (ENRICA). According to the authors, high adherence to MEDLS was inversely associated with high blood pressure. On the other hand, Romero-Cabrera et al. (33) did not find either cross-sectional or longitudinal associations between MEDLS and hypertension. In one of the few studies that examined the association with heart attack, Mata-Fernández et al. (34) found a lower risk of cardiovascular disease among participants with higher adherence to MEDLS, after analyzing data from the *Seguimiento Universidad de Navarra* (SUN) project.

Conflicting findings have also been reported for the individual components of the MEDLS. While some studies have identified significant associations between hypertension and socialization (23), others have found significant associations only for diet quality, physical activity, and sleeping patterns (22). In relation to heart attack, significance was observed for dietary components together, as well as low sedentary behavior (34).

The discrepancies between the aforementioned studies and the present study are likely attributable to several factors, including differences in population characteristics, the methods for assessing MEDLS components, the selection of covariates used to adjust the models, and the follow-up duration. Specifically, participants in ELSA constitute community-dwelling older adults, characterized by a moderate prevalence of hypertension (17.0%) and low prevalence and incidence of cardio-cerebrovascular diseases (<5%). In this context, a longer follow-up period may be required to capture a sufficient number of incident cardiovascular disease (CVD) events.

Indeed, other studies that reported significant associations between MEDLS and cardiovascular risk factors in comparable populations employed substantially longer follow-up periods (∼8 years) (32). In contrast, when a follow-up duration similar to that of the present study was used, even among participants with elevated cardiovascular risk, no significant associations were observed (33).

The low number of cardiovascular events also constrained the number of covariates that could be included in the multivariable analyses. Although the parsimonious adjustment reduced the risk of overfitting, several potentially important cardiovascular risk factors—including body mass index, smoking, diabetes, dyslipidemia, antihypertensive medication use, and educational and socioeconomic characteristics—could not be incorporated into the models. Residual confounding by these factors may therefore have influenced the observed associations and should be considered when interpreting the findings.

Another notable difference relates to the methods used to assess MEDLS components. Although most of the aforementioned studies used the validated MEDLS index, distinct approaches were adopted to assess its individual components. For instance, only moderate agreement has been observed among different MED diet indices, with the greatest discrepancies occurring at the highest levels of adherence (35). Such differences in dietary assessment may influence the classification of participants' adherence to the MED diet and, consequently, the observed associations between MED diet adherence and adverse health outcomes (36).

Findings of the present study suggest that high adherence to the MEDLS was inversely associated with hypertension, whereas no significant associations were observed for the individual MEDLS components. These results indicate that, in the examined population, the combined effect of multiple lifestyle factors, rather than any single component, may be a key determinant of the observed association.

The simultaneous adoption of healthy lifestyle behaviors has been endorsed by numerous international organizations dedicated to promoting healthy aging and cardiovascular healthy (37, 38). For example, regular physical activity and adequate nutrition have been shown to beneficially influence several pathophysiological mechanisms underlying CVD, including chronic inflammation, oxidative stress, endothelial dysfunction, and metabolic dysregulation (39–42). Likewise, social isolation—characterized by being unpartnered, having limited social participation, or maintaining a small social network—may increase CVD risk by reducing the availability of social support, delaying the recognition of health problems and healthcare seeking, and increasing psychological stress and the spread of unhealthy behaviors through social contagion (43, 44). In addition, sleep disturbances have been associated with hypertension and other CVD risk factors through mechanisms involving sympathetic nervous system activation, hypothalamic–pituitary–adrenal axis dysregulation, circadian rhythm disruption, systemic inflammation, and endothelial dysfunction (45).

However, this pattern was not observed at follow-up for incident hypertension. Instead, high adherence to the MED diet alone was significantly associated with a lower risk of heart attack. High adherence to the MED diet has long been associated with a reduced risk of CVD (11). In particular, randomized controlled trials, such as the PREDIMED trial, have demonstrated the cardiovascular benefits of a MED dietary pattern, supporting its role in the primary prevention of cardiovascular events (12).

The present study has several limitations that should be acknowledged. First, the small numbers of prevalent and incident cardiovascular events limited statistical power and precluded more comprehensive adjustment for potential confounders. Although the adjusted models included age and sex, other important cardiovascular risk factors, including body mass index, smoking, diabetes, dyslipidemia, antihypertensive medication use, and educational and socioeconomic characteristics, could not be incorporated without increasing the risk of model overfitting. Consequently, residual confounding cannot be excluded, and the observed associations should not be interpreted as independent of these factors. This represents a structural limitation of the study and restricts the robustness and generalizability of its findings. The limited number of events also resulted in sparse or empty outcome-by-exposure cells for some individual MEDLS components. Consequently, several component-specific models, particularly those involving hydration status, sleeping patterns, and cooking activities, could not be estimated reliably. The estimable component-specific associations may also have limited precision and should therefore be interpreted with caution. The low incidence of cardiovascular events may be partly attributable to the duration of follow-up, as Wave 11 was the latest available ELSA assessment.

Second, participants included in ELSA Wave 11 (2023–2024) were survivors of the COVID-19 pandemic. Although participants who experienced COVID-19-related hospitalization were excluded from the longitudinal analyses, residual confounding and selection effects associated with the pandemic cannot be ruled out.

Third, the MEDLS components were derived from self-reported measures and were therefore susceptible to recall and reporting bias, particularly among older adults. Similarly, new physician diagnoses were self-reported. These diagnoses were not verified through linked hospital or other clinical records. Participants may not have accurately recalled the timing of a diagnosis or may have been unaware of conditions that had not yet been clinically identified. Consequently, some degree of outcome misclassification cannot be excluded. Furthermore, participants who experienced a fatal stroke or heart attack during follow-up would not have been available to report a new physician diagnosis at the subsequent assessment and would therefore not have been identified as incident cases. This may have resulted in an underestimation of the incidence of these outcomes and influenced the observed longitudinal associations.

Fourth, the distributions of some MEDLS components indicate potential limitations in their classification thresholds and discriminative capacity. The social isolation criterion may have been overly sensitive, potentially overclassifying participants who reported feeling isolated only “some of the time.” Conversely, the very low prevalence of adequate hydration and the very high prevalence of adequate sleep produced marked floor and ceiling effects, respectively. This limited variability may have reduced the ability of these components to discriminate between participants and their contribution to the composite MEDLS score and component-specific associations. In addition, sleep was self-reported for a single night, which may not represent habitual sleeping patterns, while hydration was assessed exclusively from water intake because information on total fluid consumption was unavailable.

Fifth, the most appropriate method for assessing and representing physical activity remains a matter of debate, given the need to consider feasibility, reproducibility, and cost, among other factors (46). In the present study, physical activity was categorized using a dichotomous approach. Different results might have been obtained using alternative assessment or analytical approaches. Future studies using databases that include both objective and subjective measures of physical activity could examine whether associations differ by the assessment method. Such data were unavailable in the ELSA waves analyzed in the present study.

Sixth, multiple associations were examined across the overall MEDLS, its individual components, and three cardiovascular outcomes without adjustment for multiple comparisons. This increases the probability of type I error. Given the small number of statistically significant findings and the borderline statistical significance of the association between Mediterranean diet adherence and heart attack, the possibility that this result represents a chance finding cannot be excluded. This association should therefore be regarded as exploratory and hypothesis-generating, and it requires confirmation in adequately powered studies employing appropriate control for multiple testing.

Seventh, although male participants reported higher consumption of certain food groups than female participants, the limited sample size and unequal distribution across sex subgroups precluded a robust analysis of sex differences in dietary intake. Future studies with larger and more balanced samples are needed to investigate these differences.

Finally, the cross-sectional association between MEDLS and hypertension is susceptible to reverse causation. Participants with previously diagnosed hypertension may have modified their dietary or lifestyle behaviors following medical advice; therefore, the temporal direction of this association cannot be established.

At the component level, greater adherence to the Mediterranean diet was prospectively associated with lower odds of heart attack. However, given the absence of adjustment for multiple comparisons, this finding should be considered exploratory and requires confirmation in adequately powered studies.

## Data Availability

The datasets presented in this study can be found in online repositories. The names of the repository/repositories and accession number(s) can be found at https://www.elsa-project.ac.uk/.
